# Drug Eruptions

**Published:** 1887-08

**Authors:** 


**Drug Eruptions.** A Clinical Study of the Irritant Effects of Drugs upon the Skin. BY PRINCE A. MORROW, A. M., M. D., Clinical Professor of Venereal Diseases. New York: Wm. Wood & Co. 1887.

The abnormal phases of drug action are comparatively an unexplored field, and this work does much to make the special knowledge upon the subject accessible to the profession. The changes in the skin caused by drugs are sometimes strikingly similar to the outbreak of the eruptive fevers and other idiopathic affections of the skin, and the subject is, therefore, of practical interest to every physician.

